# Telerehabilitation After Surgery in Adolescent Idiopathic Scoliosis: A Randomized Controlled Trial

**DOI:** 10.3390/healthcare13162063

**Published:** 2025-08-20

**Authors:** İrem Çetinkaya, Tuğba Kuru Çolak, Mehmet Fatih Korkmaz, Mehmet Aydoğan

**Affiliations:** 1 Faculty of Health Sciences, Department of Physiotherapy and Rehabilitation, Haliç University, 34060 İstanbul, Turkey; iremkaranki@gmail.com; 2 Institute of Health Sciences, Department of Physiotherapy and Rehabilitation, Marmara University, 34899 İstanbul, Turkey; 3 Faculty of Health Sciences, Department of Physiotherapy and Rehabilitation, Marmara University, 34899 İstanbul, Turkey; 4 Institute of Health Sciences, Department of Anatomy, İstanbul Medipol University, 34726 İstanbul, Turkey; dr_mfatih@yahoo.com; 5 Advanced Spine Surgery Center, Department of Orthopedic Surgery and Traumatology, Emsey Hospital, 34912 İstanbul, Turkey; ortospine@yahoo.com

**Keywords:** scoliosis, adolescent idiopathic scoliosis, telerehabilitation, scoliosis surgery, rehabilitation

## Abstract

**Background**: Structured postoperative rehabilitation is not routinely provided for individuals with adolescent idiopathic scoliosis (AIS) after surgery, with physiotherapy typically limited to the immediate inpatient period. Telerehabilitation offers an accessible and supervised option to address persistent functional limitations, pain, and quality-of-life concerns in this population. **Objectives**: This study aimed to evaluate the effects of a synchronous telerehabilitation program—designed to support post-surgical recovery in individuals with adolescent idiopathic scoliosis (AIS)—on trunk muscle endurance, trunk flexibility, functional capacity, pain severity, perception of appearance, and quality of life. **Methods**: Thirty-two individuals with AIS, who had undergone surgery 6 months to 2 years prior, were randomly assigned to either an intervention group or a control group. The intervention group participated in a supervised telerehabilitation program twice weekly for eight weeks, while the control group received no exercise intervention. All outcome measures were assessed before and after the intervention. **Results**: The telerehabilitation group demonstrated significant improvements across all outcome measures compared with the control group (*p* < 0.05). Post-intervention, the telerehabilitation group had superior trunk muscle endurance, flexibility, and quality-of-life scores, as well as reduced pain intensity (*p* < 0.05). However, no significant differences were observed between the groups in functional capacity or perception of appearance (*p* > 0.05). **Conclusions**: A supervised telerehabilitation program initiated six months after surgery can effectively improve trunk muscle endurance, flexibility, pain intensity, and quality of life in individuals with AIS. These findings emphasize the value of structured post-surgical rehabilitation and raise awareness of the potential benefits of remotely delivered exercise programs in this population.

## 1. Introduction

The posterior spinal fusion and instrumentation (PSFI) technique, considered the gold standard for adolescent idiopathic scoliosis surgery, aims to halt curve progression, correct deformity, and achieve a balanced spine [1,2]. However, postoperative issues such as reduced spinal flexibility, decreased trunk muscle endurance, diminished functional capacity, and increased pain incidence are common, often requiring effective pain management [3,4,5,6]. Postoperative rehabilitation supervised by a physiotherapist is recommended to address these challenges and improve outcomes [7,8,9].

Telerehabilitation, a telehealth method enabling remote rehabilitation via telecommunication technologies, has shown results comparable to traditional face-to-face methods, with benefits including improved treatment adherence and patient satisfaction [10,11]. In orthopedic populations, such as patients undergoing total knee arthroplasty, hip replacement, or shoulder surgery, telerehabilitation has been shown to improve pain, function, and access to care, often matching or exceeding the results of in-person approaches [12,13]. Despite these encouraging findings, evidence for its application in post-surgical AIS rehabilitation remains scarce.

The COVID-19 pandemic further underscored the importance of remote rehabilitation, as travel restrictions and clinic closures limited access to specialized care, particularly for post-surgical AIS patients living far from treatment centers [14,15]. Therefore, implementing safe and effective remotely supervised treatment programs during the post-surgical period could help prevent and mitigate secondary complications and functional limitations following scoliosis surgery.

Therefore, the aim of this study was to investigate the effects of a synchronously delivered remote rehabilitation program tailored to the postoperative recovery of AIS patients. The primary outcomes were pain intensity and trunk muscle endurance, with secondary outcomes including flexibility, functional capacity, self-image perception, and quality of life. We hypothesized that the program would result in significant improvements in these measures, particularly in trunk muscle endurance.

## 2. Materials and Methods

### 2.1. Study Design and Participants

Patients diagnosed with AIS who underwent PSFI surgery at Emsey Hospital or Prof. Dr. Süleyman Yalçın City Hospital between 6 months and 2 years prior and attended routine follow-up visits were invited to participate in this study. This randomized, parallel-group controlled trial aimed to evaluate the superiority of a synchronous telerehabilitation program compared with no intervention. This study adhered to the CONSORT guidelines and TIDieR checklist. Ethical approval was obtained from the Haliç University Clinical Research Ethics Committee (24.06.2021/130), and the protocol was registered at ClinicalTrials.gov (NCT05669859). All patients were informed about this study, and written informed consent was obtained.

The exclusion criteria included previous spinal surgery, severe neuromuscular, rheumatologic, or orthopedic diseases, congenital deformities, significant psychiatric or psychological disorders, cognitive or communication impairments, and participation in any other rehabilitation program. Participants were randomly assigned to either the telerehabilitation group or the control group.

### 2.2. Sample Size and Randomization

Power analysis was conducted using the G*Power 3.1.9.7 software [16]. As there are no existing studies on postoperative telerehabilitation in AIS, the effect size (0.9) was estimated based on a previous study [8] involving a post-surgical endurance training program in a similar population. A sensitivity analysis using a more conservative effect size indicated that a substantially larger sample size would be required. Given the scope and resources of the present study, the target sample size was set at 32 participants (16 per group), providing 80% power at a 95% confidence level.

In this randomized controlled trial, the two groups were created using a computer-generated simple randomization method (1:1 allocation) via random.org. No restrictions such as stratification or blocking were applied. Group assignments were implemented using a sealed envelope method: upon arrival at the hospital, participants selected opaque, sequentially numbered envelopes. The researcher responsible for enrollment was blinded to the allocation sequence, ensuring allocation concealment.

### 2.3. Telerehabilitation Group

The telerehabilitation group received an online rehabilitation program designed by the researchers, conducted twice a week for a total duration of 8 weeks (Appendix A). The intervention duration and frequency were selected based on previous post-surgical rehabilitation studies reporting clinically meaningful improvements with programs lasting 6–12 weeks with 1–3 sessions per week, and to ensure feasibility and adherence in adolescents with academic commitments [17,18,19]. Each exercise session lasted approximately one hour.

Once spinal stability was achieved and fusion was completed—typically within three months post-surgery—exercises targeting spinal stabilization and mobility were recommended, with dynamic stabilization exercises initiated from the sixth month onward [20,21,22]. The rehabilitation program in this study focused on enhancing core muscle control and promoting dynamic spinal stabilization, which are essential for these patients. All exercises incorporated controlled breathing techniques and emphasized extremity mobility as well as the activation of multiple muscle groups.

On the initial evaluation day, the telerehabilitation group attended a one-hour, face-to-face training session conducted by the physiotherapist responsible for delivering the exercise program. During this session, participants received both verbal and written information regarding the anatomy and biomechanics of muscles involved in stabilizing the spine and pelvis, with particular emphasis on the multifidus, transversus abdominis, diaphragm, and pelvic floor muscles. Key elements of the exercise program—such as maintaining focus during deep trunk muscle contractions, diaphragmatic breathing, and respiratory control during exercises—were explained. In addition, primary exercise positions and basic stretching techniques were demonstrated.

Each exercise session began with a five-minute warm-up and concluded with a five-minute cool-down. The complete content of the eight-week rehabilitation program is provided in Appendix A.

### 2.4. Control Group

The control group, which attended routine radiological evaluations and physical examinations at six-month to one-year intervals post-surgery without participating in any rehabilitation program, was informed—during the consent process—that they could opt to receive the telerehabilitation program after the 8-week control period. This approach was implemented to address potential disappointment bias and to ensure ethical fairness between groups. In our country, no structured rehabilitation program is typically provided after discharge from intensive care or hospitalization for scoliosis surgery. Therefore, this group represents the standard care pathway and served as the control group in this study.

Due to the nature of the intervention, blinding of participants and assessors was not feasible, as the exercise group attended supervised telerehabilitation sessions, while the control group received no intervention. To minimize potential bias, all assessments were performed by the same physiotherapist using standardized protocols.

### 2.5. Measurements

Demographic and clinical characteristics were recorded. All patients were assessed at two time points—before and after the intervention—separated by an 8-week interval. Post-surgical spinal curvature was measured using the Cobb method, based on standing X-rays taken one week prior to the evaluation.

Trunk muscle endurance was assessed using McGill’s position maintenance tests. Trunk flexor endurance was measured with the Kraus–Weber test [23], extensor endurance with the Biering–Sørensen test [24], and anterior, posterior, and lateral stabilizer endurance with the Side Bridge test [25]. The duration for which each position was maintained was recorded in seconds using a stopwatch.

Thoracolumbar flexibility was assessed using the Sit-and-Reach test. The patient sat with legs extended and both elbows and knees fully extended, and was instructed to reach forward toward the feet with their arms. The distance between the third phalanx and the toes was measured in centimeters (cm). A negative value was recorded if the fingers did not reach the toes, and a positive value if they extended beyond them [26,27].

Trunk lateral flexibility was assessed using the Lateral Bending test. The patient stood with their back against the wall and arms resting at their sides. The distance from the third phalanx to the floor was measured at rest and again during a side bend. The difference between these two values was recorded in centimeters (cm), and the procedure was repeated for the opposite side [26,27]. Each flexibility test was performed three times, and the average of the three measurements was used for analysis.

The Six-Minute Walk Test was used to assess patients’ functional capacity. Participants walked at a self-selected pace along a 30 m corridor for six minutes. The total distance covered was recorded in meters based on the number of laps completed [28].

Pain intensity was assessed using the Numeric Rating Scale (NRS), where patients rated their pain on a scale from 0 to 10 (0 = no pain; 10 = unbearable pain) [29].

Patients’ perceptions and expectations regarding spinal appearance were assessed using the Spinal Appearance Questionnaire (SAQ), a valid and reliable instrument with established Turkish validity and reliability in individuals with AIS. Higher scores indicate more negative perceptions and greater concerns about appearance [30].

Quality of life in patients who underwent scoliosis surgery was evaluated using the Scoliosis Research Society Questionnaire-30 (SRS-30), a valid and reliable instrument with Turkish validity and reliability established in individuals with AIS. The questionnaire comprises five subscales: pain, function, self-image, mental health, and satisfaction. Higher scores reflect a better quality of life [31].

### 2.6. Statistical Analysis

Data analysis was performed using IBM SPSS Statistics Version 24 (SPSS Inc., Chicago, IL, USA). Continuous variables were presented as means ± standard deviations or medians (minimum–maximum), while categorical variables were presented as counts and percentages (%). The chi-square test was used to compare categorical variables between groups. The Shapiro–Wilk test assessed data normality. For normally distributed variables, the independent-samples *t*-test and the paired-samples *t*-test were used, while for non-normally distributed variables, the Mann–Whitney U test and the Wilcoxon signed-rank test were applied. Effect sizes were calculated alongside *p*-values, using Cohen’s d for parametric tests and the effect size r for non-parametric tests, each with corresponding 95% confidence intervals (95% CIs) to aid interpretation. Cohen’s d-values were interpreted as negligible (<0.20), small (0.20–0.50), moderate (0.50–0.80), large (0.80–1.30), or very large (>1.30) [32]. Effect size r-values were interpreted as negligible (<0.10), small (0.10–0.30), moderate (0.30–0.50), or large (>0.50) [33]. Confidence intervals of 95 percent (95% CIs) were reported for all effect sizes. Statistical significance was set at a two-sided *p*-value of ≤0.05.

## 3. Results

**Figure 1:**
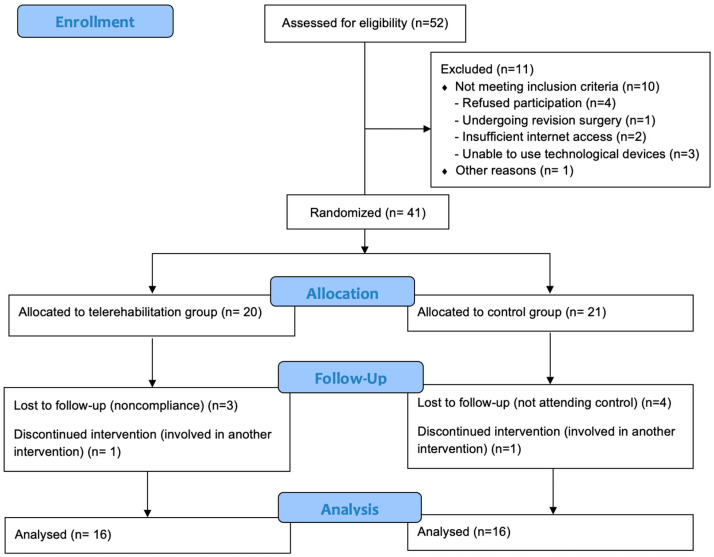
Flow diagram of this study.

A total of 32 participants were included in this study (Figure 1), with 16 in the telerehabilitation group (TG) and 16 in the control group (CG). The baseline demographic and clinical characteristics were similar between the groups (*p* > 0.05) (Table 1).

All patients included in this study had undergone posterior spinal fusion surgery. Following the operation, all patients were advised to use a brace, with the mean duration of brace use being three months in both groups. The purpose of brace use was to provide additional trunk support, maintain proper spinal alignment during the healing process, and reduce mechanical stress on the surgical site.

The mean age of the patients in both groups was over 17 years, and their Risser scores were above 4, indicating that skeletal growth was largely complete. A total of 53.1% of the patients had a major thoracic curvature. The majority of patients (71.8%) had not received any physiotherapy for scoliosis prior to surgery, and only a small proportion (9.3%) reported regular participation in sports activities. In the TG, fused segments ranged from T3 to L4; in the CG, from T2 to L4, with no significant difference in the number of fused vertebrae between the groups (*p* = 0.924).

There were no statistically significant differences between the groups at baseline in trunk endurance, trunk flexibility, pain intensity, functional capacity, perception of appearance, or quality of life scores (*p* > 0.05) (Table 2). After the intervention, the telerehabilitation group showed significant improvements in their trunk endurance and flexibility scores, Six-Minute Walk Test results, pain intensity, perception of appearance, and quality-of-life scores (*p* < 0.001). However, no significant between-group differences were observed in functional capacity, perception of appearance, self-image, and mental health subscale scores after treatment (*p* > 0.05) (Table 2).

When comparing pre- with post-treatment change scores, the TG achieved significantly greater improvements than the CG across all outcome measures (*p* < 0.05), with particularly pronounced gains in trunk endurance (e.g., +29 s vs. +0.6 s in the Biering–Sørensen test, and +24 s vs. +5.3 s in the Kraus–Weber test) and flexibility (e.g., +4.1 cm vs. −0.1 cm in the Sit-and-Reach test) (Table 3).

## 4. Discussion

In the current study, telerehabilitation was found to be effective in improving trunk muscle endurance, trunk flexibility, and functional capacity, reducing pain intensity, and enhancing perceptions of appearance and quality of life in patients with AIS after surgical treatment.

Trunk muscle endurance has been reported to decrease after scoliosis surgery, often linked to reduced physical function and increased back pain [7,34]. In this study, participants showed low trunk muscle endurance compared with normative values [18,35]. Therapeutic exercises like the Schroth method and core stabilization have been effective in improving endurance in AIS patients [36,37]. However, no study has specifically targeted trunk muscle endurance in the fused spine during the post-surgical period. Training the trunk’s core muscles, which work synergistically for stabilization, is critical in rehabilitation. The transversus abdominis (TrA), forming a musculofascial corset, plays a vital role in symmetrical contraction cycles and trunk stabilization during static and dynamic exercises [38,39].

Our eight-week exercise program in this study significantly improved trunk flexor, extensor, and lateral muscle endurance, with post-intervention endurance in the TG exceeding the CG. Patients were taught proper TrA activation to ensure core muscle engagement, and exercises emphasized symmetrical, dynamic patterns. Improvements likely stemmed from integrating key principles, patient education, and exercises targeting local and global core systems, such as bridge exercises with breathing control.

While the primary goal of surgical treatment is maximum curve correction, patient functionality should not be overlooked [40]. In PFE surgery, spinal fusion creates a rigid column to control asymmetric growth [41], but it reduces trunk mobility and flexibility, especially with distal fusion [7,42,43]. Post-surgery, patients may adopt protective movement strategies, further limiting non-fused regions and increasing functional restrictions [41,44]. Movement anxiety and trunk avoidance behaviors can also exacerbate these limitations, reducing daily activities and flexibility.

In this study, trunk flexibility significantly improved in the TG, with results and change values surpassing the CG. These improvements likely stemmed from dynamic patterns and flexibility exercises targeting trunk and extremity muscle extensibility, incorporated at the end of each session and during repetition days.

Two studies have evaluated the effects of in-hospital and post-discharge rehabilitation on functional capacity during the postoperative period. In an in-hospital study, the Two-Minute Walk Test showed significant improvement in functional capacity after a five-day physiotherapist-supervised program [45]. Laurentowska et al. reported that a four-week aerobic-based rehabilitation program, administered 1 to 3 years post-surgery, significantly increased exercise capacity in AIS patients [8].

Consistent with these findings, our study showed a significant increase in functional capacity in TG after treatment. This improvement may be attributed to the controlled breathing integrated into our telerehabilitation exercises. However, no significant difference between groups was found, likely because baseline measurements were similar to healthy reference values and our program emphasized stabilization over aerobic training.

Low sports participation was noted among our participants, with only 3 out of 32 reporting regular sports habits, often due to fear of damaging surgical materials or needing supervision. Shen et al. highlighted better functional capacities in AIS patients with regular exercise habits [46], while Sperandio et al. suggested that poor breathing patterns during exercise may contribute to avoidance behavior [47]. The greater functional capacity improvement in TG may be linked to their adoption of regular exercise habits during the program.

Although evidence on the long-term effects of surgical treatment on spinal pain in AIS is limited, studies report varying outcomes: pain reduction, no change, or even increased chronic pain [2,48,49,50]. Weiss applied an intensive pain management-focused inpatient rehabilitation program to patients with chronic pain at least 10 years post-surgery, showing decreased chronic pain and highlighting the need for further postoperative rehabilitation studies [7].

Only one study has examined pre-discharge rehabilitation after scoliosis surgery, reporting reduced perceived pain intensity following an in-hospital program [45]. Similarly, the current study showed a significant reduction in patient-reported pain after the telerehabilitation program, with post-intervention pain scores in the TG significantly lower than in the CG. These improvements may be attributed to enhanced trunk control and regular physical activity through systematically progressive physiotherapy exercises.

Although instrumented fusion surgeries reduce curve severity, postoperative expectations and body image perception significantly influence treatment success [51,52]. AIS patients ideally expect a straight spine, balanced shoulders, and an appearance without a hump after surgery [53]. However, achieving optimal self-image perception is often challenging. One study reported that only 27% of surgically treated patients had a positive body image perception [54], while another found that adults with IS who underwent surgery had more negative views on their appearance than those untreated [55].

No study has yet examined the impact of postoperative rehabilitation on body image perception in AIS. In our study, telerehabilitation significantly improved body image perception within and between groups based on change averages. This may be attributed to enhanced endurance and flexibility through exercise, promoting postural alignment and positive awareness. However, the similarity between groups post-treatment may reflect the influence of physiological, sociocultural, and biopsychological factors on adolescents’ perceptions.

While deformity reduction after AIS surgery may improve quality of life, factors such as visible scarring, postoperative pain, reduced spinal flexibility, and activity limitations can negatively impact functionality and quality of life [6,48,56,57,58]. Patient-reported quality of life has become a key indicator of surgical success, emphasizing the importance of patient-centered care alongside traditional radiographic outcomes [51,59,60]. Additionally, health economics is significantly influenced by postoperative satisfaction and care processes, making strategies to enhance surgical outcomes and patient satisfaction critical [61].

In this study, telerehabilitation improved the quality of life and all subgroups, with significantly greater improvements in total and subgroup scores compared with the control group. Participants in the telerehabilitation group experienced less pain and higher functional and treatment satisfaction levels, whereas quality of life decreased and pain-related dissatisfaction increased in the control group. Despite significant within-group improvements, no between-group differences were observed in self-image and mental health subgroup outcomes post-intervention. These findings may reflect the multifactorial nature of these constructs, which are influenced not only by physical rehabilitation but also by social, cultural, and psychological factors that may not be fully addressed by exercise-based interventions alone. Additionally, the relatively short intervention period may have limited the potential for observable differences between groups. Future research could explore the integration of psychosocial support and longer-term interventions to address these outcomes more comprehensively.

To the best of our knowledge, this is the first randomized controlled trial implementing a rehabilitation program in the post-discharge period after scoliosis surgery, aimed at supporting adaptation and recovery of the fused spine while preventing potential secondary complications. A limitation of this study is the lack of long-term follow-up and the inability to compare the outcomes of telerehabilitation with those of in-person rehabilitation programs. Another limitation is the potential for selection bias, as participation in the telerehabilitation group was voluntary and may have attracted patients who were more motivated or more comfortable with technology. This could have influenced adherence and outcome measures.

The findings of this study suggest that structured, synchronously delivered telerehabilitation programs can be effectively implemented in postoperative AIS patients, even in settings with limited access to in-person rehabilitation. Such programs could be particularly beneficial in rural or underserved areas, where travel to specialized spine centers is challenging. By reducing geographic and logistical barriers, telerehabilitation may facilitate earlier initiation and greater continuity of care, potentially improving long-term functional and quality-of-life outcomes.

## 5. Conclusions

This study highlights the potential of structured postoperative rehabilitation, such as telerehabilitation, to improve outcomes for individuals with AIS following surgical treatment. In our cohort, telerehabilitation was associated with significant gains in trunk muscle endurance, flexibility, body image perception, and quality of life, along with reduced pain, enhanced functional capacity, and improved patient satisfaction and self-awareness. As a remotely delivered and potentially scalable intervention, telerehabilitation may help address physical, psychological, and sociocultural challenges faced by AIS patients, particularly in regions with limited access to specialized in-person rehabilitation. However, confirmation of these benefits in larger, multi-center trials with longer follow-up is warranted before definitive conclusions can be drawn.

## Figures and Tables

**Table 1:** Baseline characteristics of the participants.

Characteristics	TG (n = 16)	CG (n = 16)	*p*
Age (years, mean ± SD)	17.38 ± 2.36	17.44 ± 2.07	0.937 ^a^
Gender (female/male, %)	13/3, (81.3/18.8)	10/6, 62.5/37.5	0.433 ^b^
Height (m, mean ± SD)	166.75 ± 9.46	169.38 ± 8.85	0.424 ^a^
Weight (kg, mean ± SD)	56.81 ± 8.04	56.19 ± 8.69	0.834 ^a^
BMI (kg/m^2^, mean ± SD)	20.47 ± 2.5	19.64 ± 2.85	0.383 ^a^
PTR history (yes/no, %)	5/11, (31.3/68.8)	4/12, (25/75)	1.000 ^b^
Sports habit (yes/no, %)	1/15 (6.3/93.8)	2/14 (12.5/87.5)	1.000 ^b^
Scoliosis classification (T, TL, L, %)	9/4/3, 56.3/25/18.8	8/6/2 (50/37.5/12.5)	0.796 ^b^
Risser stage (mean ± SD)	4.44 ± 0.81	4.69 ± 0.6	0.299 ^c^
Preop. Cobb angle (°, mean ± SD)	55 ± 14.22	55.25 ± 9.28	0.953 ^a^
Postop. Cobb angle (°, mean ± SD)	12.31 ± 5.85	9.88 ± 4.97	0.273 ^c^
Number of fused vertebrae (mean ± SD)	11.69 ± 2.5	11.63 ± 2.91	0.924 ^c^
Time since surgery (months, mean ± SD)	16.81 ± 6.12	16 ± 5.89	0.592 ^c^
Post-surgical brace duration (months, mean ± SD)	3.25 ± 2.08	2.69 ± 1.7	0.469 ^c^

SD: standard deviation, m: meter, kg: kilogram, BMI: body mass index, PTR: physiotherapy and rehabilitation, T: thoracic, TL: thoracolumbar, L: lumbar, TG: telerehabilitation group, CG: control group, a: independent-samples *t*-test, b: Fisher’s exact test, c: Mann–Whitney U test, and statistically significant: *p* < 0.05.

**Table 2:** Outcome measures between groups and within groups.

		TG (n = 16) Mean ± SD/Median (Min.–Max.)	CG (n = 16) Mean ± SD/Median (Min.–Max.)	*p* (Between-Group)	d/r [95% CI] (Between-Group)
Kraus–Weber test (sec)	Pre-treatment Post-treatment p (within-group) d/r [95% CI]	42.88 ± 35.16/31 (6–124) 67 ± 34.89/59 (27–148) **<0.001 ^c^** −0.88 [−0.96−(−0.68)]	35.75 ± 28.36/30.5 (6–105) 41.06 ± 35.32/26.5 (12–96) 0.103 ^c^ −0.41 [−0.75–0.11]	0.546 ^b^ **0.013 ^b^**	−0.44 [−0.68–(−0.11)]
Biering–Sørensen test (sec)	Pre-treatment Post-treatment p (within-group) d/r [95% CI]	12.13 ± 14.72/7 (1–55) 41.44 ± 30.13/26.5 (12–96) **<0.001 ^c^** −0.87 [0.95−(−0.68)]	15.25 ± 14.51/12 (1–52) 15.81 ± 13.31/13 (1–43) 0.529 ^c^ −0.16 [−0.61–0.37]	0.427 ^b^ **0.005 ^b^**	−0.49 [−0.72–(−0.18]
Lateral Bridge test–left (sec)	Pre-treatment Post-treatment p (within-group) d/r [95% CI]	16.5 ± 14.02/15 (1–45) 31.94 ± 18.16/28 (9–62) **<0.001 ^d^** −1.48 [−20.1−(−9.88)]	16.19 ± 14.99/13.5 (1–62) 18.94 ± 16.92/16 (1–67) 0.074 ^c^ −0.45 [−0.77–0.06]	0.94 ^b^ **0.035 ^b^**	−0.37 [−0.64–(−0.03)]
Lateral Bridge test–right (sec)	Pre-treatment Post-treatment p (within-group) d/r [95% CI]	14.94 ± 12.32/12 (1–36) 31.75 ± 15.17/31 (10–57) **<0.001 ^c^** −0.88 [−0.96−(−0.68)]	16.13 ± 12.14/15 (1–47) 16.31 ± 12.74/15 (1–50) 0.864 ^d^ −0.04 [−2.48–2.1]	0.691 ^b^ **0.004 ^a^**	1,1 [5.31–25.57]
Sit-and-Reach test (cm)	Pre-treatment Post-treatment p (within-group) d/r [95% CI]	−22.06 ± 11.67/−23 (−38−[−1]) −17.88 ± 11.67/−16.5 (−35–5) **<0.001 ^d^** −1.91 [−5.36−(−3.02)]	−25.75 ± 9.87/−25 (−46−[−1]) −25.88 ± 9.34/−26 (−44−[−1]) 0.827 ^d^ −0.06 [−1.07–1.32]	0.342 ^a^ **0.041 ^a^**	0.76 [0.35–15.65]
Lateral Bending test–left (cm)	Pre-treatment Post-treatment p (within-group) d/r [95% CI]	9.88 ± 3.4/10 (5–15) 11.88 ± 3.44/11.5 (6–17) **<0.001 ^d^** −1.73 [−2.61−(−1.39)]	8.94 ± 2.2/8.5 (6–13) 9.25 ± 2.52/8.5 (6–14) 0.173 ^d^ −0.36 [−0.78–0.15]	0.362 ^a^ **0.02 ^a^**	0.87 [0.44–4.81]
Lateral Bending test–right (cm)	Pre-treatment Post-treatment p (within-group) d/r [95% CI]	9.75 ± 3.66/8.5 (5–15) 11.63 ± 3.78/11.5 (7–18) **0.001 ^c^** −0.83 [−0.94−(−0.57)]	8.5 ± 2.39/9 (4–12) 8.81 ± 2.43/8.5 (5–13) 0.333 ^d^ −0.24 [−0.98–0.35]	0.414 ^b^ **0.018 ^a^**	0.89 [0.5–5.12]
Six-Minute Walk Test (m)	Pre-treatment Post-treatment p (within-group) d/r [95% CI]	544.75 ± 64.86/531.5 (465–690) 565.38 ± 58.56/553.5 (495–685) **<0.001 ^c^** −0.79 [−0.92−(−0.49)]	557.56 ± 55.19/527.5 (502–675) 556.13 ± 56.59/533.5 (483–665) 0.842 ^c^ −0.05 [−0.53–0.45]	0.365 ^b^ 0.598 ^b^	−0.09 [−0.43–0.26]
Numeric Rating Scale	Pre-treatment Post-treatment p (within-group) d/r [95% CI]	4.69 ± 1.96/5 (1–7) 1.94 ± 0.998/2 (1–4) **0.001 ^c^** −0.86 [−0.95−(−0.64)]	3.94 ± 1.95/4 (1–7) 4.13 ± 2.13/5 (1–7) 0.594 ^d^ −0.13 [−0.92–0.55]	0.286 ^a^ **0.004 ^b^**	−0.51 [−0.73−(−0.19)]
SAQ (total)	Pre-treatment Post-treatment p (within-group) d/r [95% CI]	30 ± 8.59/30 (16–47) 25.31 ± 5.84/26 (15–38) **<0.001 ^d^** 1.24 [2.67–6.71]	28.19 ± 9.04/31 (15–40) 28.94 ± 9.18/29 (17–47) 0.583 ^d^ −0.14 [−3.6–2.1]	0.565 ^a^ 0.193 ^a^	−0.47 [−9.18–1.93]
SAQ (appearance)	Pre-treatment Post-treatment p (within-group) d/r [95% CI]	17.63 ± 4.43/17 (12–28) 15.13 ± 2.96/15 (11–22) **<0.001 ^d^** 1.13 [1.32–3.69]	16.69 ± 4.62/16.5 (10–24) 17.13± 5.37/16.5 (10–30) 0.578 ^d^ −0.14 [−2.08–1.2]	0.562 ^a^ 0.202 ^a^	−0.46 [−5.13–1.13]
SAQ (expectation)	Pre-treatment Post-treatment p (within-group) d/r [95% CI]	12.38 ± 6.58/15.5 (4–20) 10.19 ± 4.45/10.5 (4–16) **0.01 ^c^** −0.64 [−0.86−(−0.22)]	11.5 ± 5.42/12.5 (4–20) 11.81 ± 5.33/13 (5–20) 0.739 ^d^ −0.08 [−2.28–1.65]	0.531 ^b^ 0.357 ^a^	−0.33 [−5.17–1.92]
SRS-30 (total)	Pre-treatment Post-treatment p (within-group) d/r [95% CI]	19.08 ± 2.44 /19 (15–23.8) 21.68 ± 1.41/ 21.4 (19.1–24.1) **<0.001 ^d^** −1.73 [−3.4−(−1.8)]	20.54 ± 2.07/20.6 (17.5–24.1) 19.99 ± 2.02/20 (16.5–23.2) **0.021 ^d^** −0.64 [0.09–0.99]	0.077 ^b^ **0.01 ^a^**	0.97 [0.42–2.94]
SRS-30 (pain)	Pre-treatment Post-treatment p (within-group) d/r [95% CI]	3.63 ± 0.75/ 3.5 (2.3–5) 4.3 ± 0.34/ 4.3 (3.8–4.8) **<0.001 ^d^** −1.19 [−0.96−(−0.37)]	3.99 ± 0.69/ 4.2 (2.8–5) 3.83 ± 0.76/ 3.9 (2.6–5) **0.033 ^d^** −0.59 [0.01–0.3]	0.179 ^a^ **0.032 ^a^**	0.8 [0.04–0.9]
SRS-30 (function)	Pre-treatment Post-treatment p (within-group) d/r [95% CI]	3.74 ± 0.66/ 3.9 (2.4–4.7) 4.38 ± 0.45/ 4.4 (3.7–5) **<0.001 ^d^** −1.56 [−0.86−(−0.43)]	4.11 ± 0.54/4 (3.3–5) 4.05 ± 0.42/4 (3.4–4.9) 0.485 ^d^ −0.18 [−0.11–0.22]	0.093 ^a^ **0.004 ^a^**	0.76 [0.02–0.65]
SRS-30 (self-image)	Pre-treatment Post-treatment p (within-group) d/r [95% CI]	4.06 ± 0.53/4 (3–5) 4.4 ± 0.47/4.5 (3.4–5) **0.015 ^d^** −0.68 [−0.59−(−0.07)]	4.21 ± 0.49/4.2 (3.4–5) 4.06 ± 0.5/4.1 (3.3–5) 0.163 ^d^ −0.37 [−0.07–0.38]	0.438 ^a^ 0.54 ^a^	0.71 [−0.06–0.69]
SRS-30 (mental health)	Pre-treatment Post-treatment p (within-group) d/r [95% CI]	3.29 ± 0.58/3.1 (2.4–4.4) 3.7 ± 0.41/3.6 (3–4.4) **0.004 ^d^** −0.85 [−0.67−(−0.15)]	3.65 ± 0.44/3.6 (3–4.4) 3.65 ± 0.6/3.7 (2.2–4.4) 1.000 ^d^ 0.0 [−0.23–0.23]	0.055 ^a^ 0.786 ^a^	0.1 [−0.32–0.42]
SRS-30 (satisfaction)	Pre-treatment Post-treatment p (within-group) d/r [95% CI]	4.33 ± 0.76/4.7 (2–5) 4.89 ± 0.21/5 (4.3–5) **0.002 ^c^** −0.77 [−0.92−(−0.45)]	4.58 ± 0.53/4.7 (3.7–5) 4.63 ± 0.42/4.7 (4–5) 0.865 ^c^ −0.04 [−0.53–0.46]	0.205 ^b^ **0.046 ^b^**	−0.35 [−0.63−(−0.005]

SD: standard deviation, d/r: effect size, CI: confidence interval, m: meter, cm: centimeter, sec: second, TG: telerehabilitation group, CG: control group, a: independent-samples *t*-test, b: Mann–Whitney U test, c: Wilcoxon signed-rank test, d: paired-samples *t*-test, and statistically significant difference: *p* < 0.05 and bold-marked.

**Table 3:** Comparison of mean changes before and after treatment between groups.

		TG (n = 16) Mean ± SD/Median (Min.–Max.)	CG (n = 16) Mean ± SD/Median (Min.–Max.)	*p*	d/r [95% CI]
Kraus–Weber test (sec)	∆ _pre-post_	24.13 ± 12.73/21 (9–66)	5.31 ± 10.24/3 (−6–29)	**<0.001 ^b^**	−0.67 [−0.83−(−0.43)]
Biering–Sørensen test (sec)	∆ _pre-post_	29.31 ± 19.61/24 (9–68)	0.56 ± 7.02/1.5 (−17–14)	**<0.001 ^b^**	−0.82 [−0.91−(−0.66)]
Lateral Bridge test–left (sec)	∆ _pre-post_	15.44 ± 10.4/10.5 (2–41)	2.75 ± 5.5/2.5 (−6–17)	**<0.001 ^a^**	1.522 [6.59–18.79]
Lateral Bridge test–right (sec)	∆ _pre-post_	16.81 ± 8.76/15 (4–32)	0.19 ± 4.29/0 (−7–9)	**<0.001 ^a^**	2.411 [11.57–21.68]
Sit-and-Reach test (cm)	∆ _pre-post_	4.06 ± 2.32/3.5 (1–9)	−0.13 ± 2.25/0 (−5–4)	**<0.001 ^a^**	1.94 [2.71–5.92]
Lateral Bending test–left (cm)	∆ _pre-post_	2 ± 1.16/2 (0–4)	0.31 ± 0.87/0.5 (−2–1)	**<0.001 ^b^**	−0.65 [−0.81−(−0.39)]
Lateral Bending test–right (cm)	∆ _pre-post_	1.88 ± 1.36/1.5 (0–4)	0.31 ± 1.25/0 (−2–3)	**0.003 ^b^**	−0.52 [−0.74−(−0.21)]
Six-Minute Walk Test (m)	∆ _pre-post_	20.63 ± 17.55/19 (−8–50)	−1.44 ± 14.77/−2.5 (−42–17)	**<0.001 ^b^**	−0.6 [−0.79−(−0.32)]
Numeric Rating Scale	∆ _pre-post_	−2.75 ± 1.39/−3 (−5–0)	0.19 ± 1.38/0 (−3–3)	**<0.001 ^b^**	−0.76 [−0.87−(−0.55)]
SAQ (total)	∆ _pre-post_	−4.69 ± 3.79/−4 (−12–1)	0.75 ± 5.35/2 (−12–9)	**0.002 ^a^**	−1.173 [−8.78–2.09]
SAQ (appearance)	∆ _pre-post_	−2.5 ± 2.22/−1.5 (−7–0)	0.44 ± 3.08/0 (−4–10)	**0.001 ^b^**	−0.61 [−0.79−(−0.33)]
SAQ (expectation)	∆ _pre-post_	−2.19 ± 3.08/−2 (−9–2)	0.31 ± 3.68/1 (−8–8)	**0.046 ^a^**	−0.736 [−4.95–0.05]
SRS-30 (total)	∆ _pre-post_	2.6 ± 1.5/2.7 (−0.7–5.2)	−0.5 ± 0.85/−0.35 (−1.8–0.7)	**<0.001 ^a^**	2.575 [2.26–4.03]
SRS-30 (pain)	∆ _pre-post_	0.66 ± 0.56/0.8 (−0.3–1.5)	−0.16 ± 0.3/−0.2 (−0.7–0.5)	**<0.001 ^a^**	1.876 [0.5–1.13]
SRS-30 (function)	∆ _pre-post_	0.64 ± 0.4/0.65 (0.1–1.6)	−0.06 ± 0.3/−0.05 (−0.7–0.5)	**<0.001 ^a^**	1.937 [0.44–0.96]
SRS-30 (self-image)	∆ _pre-post_	0.33 ± 0.48/0.4 (−0.8–1.1)	−0.15 ± 0.43/−0.15 (−0.8–0.7)	**0.005 ^a^**	1.069 [0.16–0.81]
SRS-30 (mental health)	∆ _pre-post_	0.41 ± 0.49/0.5 (−0.4–1.6)	0.0 ± 0.43/−0.1 (−0.8–0.8)	**0.017 ^a^**	0.896 [0.08–0.74]
SRS-30 (satisfaction)	∆ _pre-post_	0.57 ± 0.61/0.3 (0–2.3)	0.44 ± 0.59/0 (−0.7–1.8)	**0.001 ^b^**	−0.57 [−0.76−(−0.27)]

SD: standard deviation, d/r: effect size, CI: confidence interval, m: meter, cm: centimeter, sec: second, TG: telerehabilitation group, CG: control group, ∆ _pre-post_: change in means between pre-treatment and post-treatment measurements, a: independent-samples *t*-test, b: Mann–Whitney U test, and statistically significant difference: *p* < 0.05 and bold-marked.

**Figure A1:**
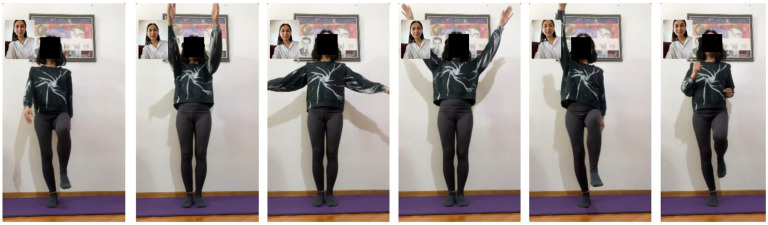
Warm-up Program.

**Figure A2:**
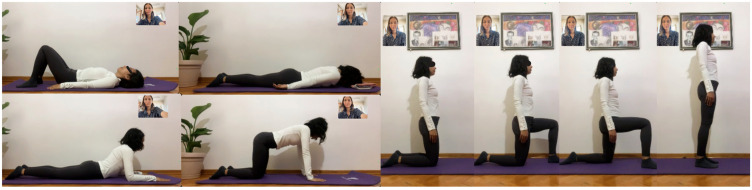
Week 1 Exercises.

**Figure A3:**
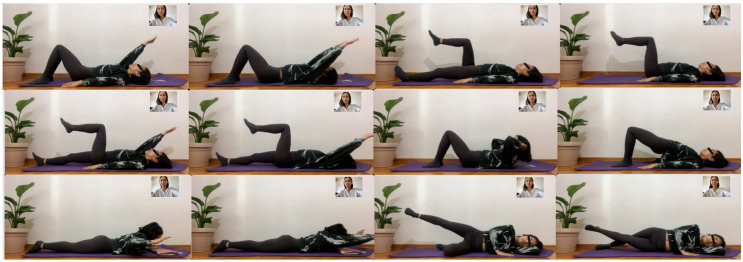
Week 2 Exercises.

**Figure A4:**
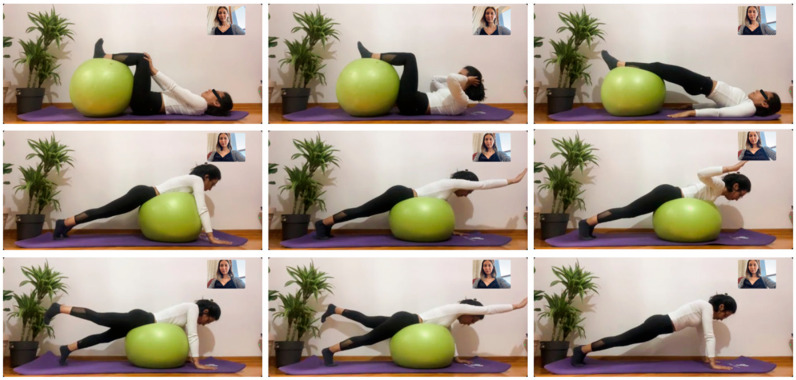
Week 3 Exercises.

**Figure A5:**
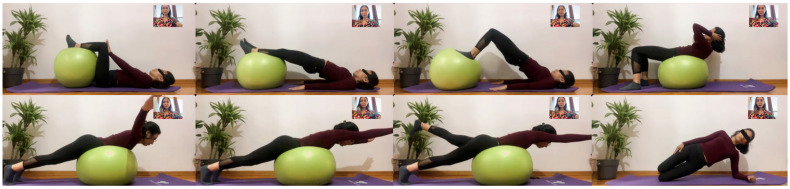
Week 4 Exercises.

**Figure A6:**
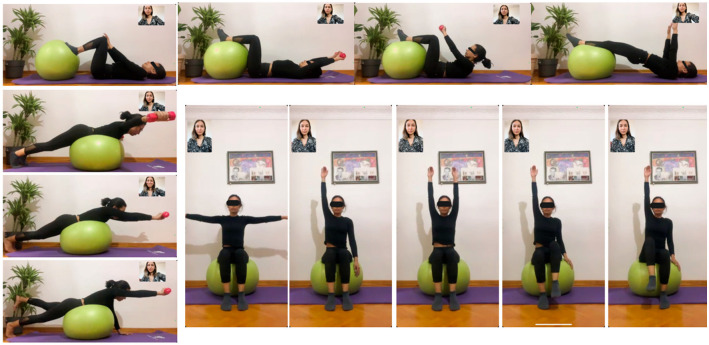
Week 5 Exercises.

**Figure A7:**
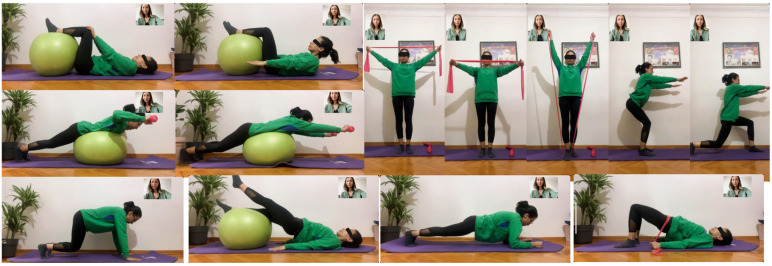
Week 6 Exercises.

**Figure A8:**
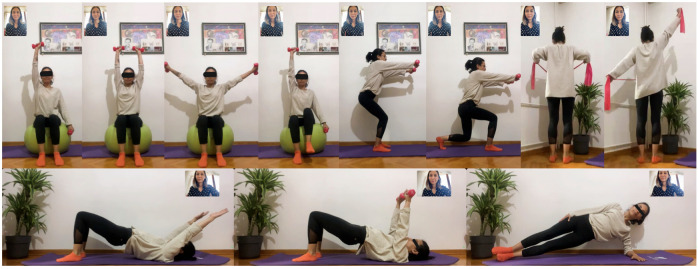
Week 7 Exercises.

**Figure A9:**
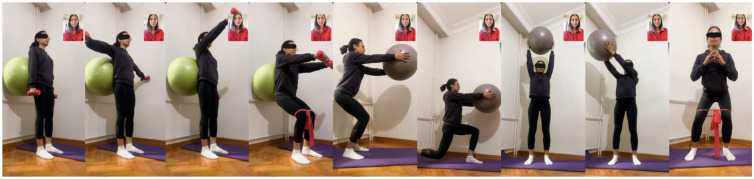
Week 8 Exercises.

**Figure A10:**
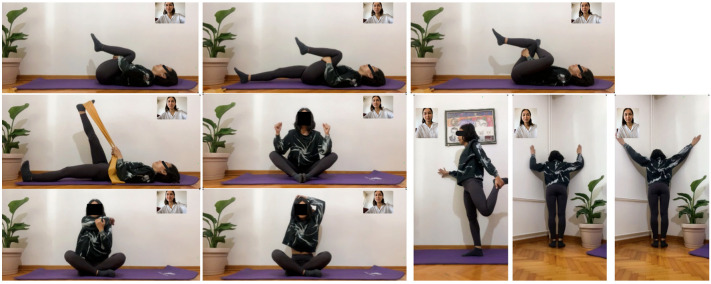
Cool-down program.

## Data Availability

The data that support the findings of this study are not publicly available due to privacy or ethical restrictions involving personal or patient information. Other relevant data are available from the corresponding author upon reasonable request.

## Abbreviations

The following abbreviations were used in this manuscript:

AIS	Adolescent Idiopathic Scoliosis
BMI	Body Mass Index
CG	Control Group
L	Lumbar
PSFI	Posterior Spinal Fusion and Instrumentation
PTR	Physiotherapy and Rehabilitation
SAQ	Spinal Appearance Questionnaire
SRS-30	Scoliosis Research Society-30
T	Thoracic
TG	Telerehabilitation Group
TL	Thoracolumbar
TrA	Transversus Abdominis

## Appendix A. Exercise Protocol

*Warm-up Program:*

Each session began with a general warm-up program of approximately five minutes (Figure A1):

- Step counting in place;
- Lifting arms forward, sideways, and diagonally while counting steps in place;
- Step counting with reciprocal lower- and upper-extremity movements;
- Soldier march;
- Jogging in place at a light pace.

*Week 1 Exercises.*

Warm-up program
Core muscle activation consisting of 8 s isometric contractions together with diaphragmatic breathing while maintaining the neutral position of the spine and pelvis was applied in the static positions specified below (Figure A2). Core muscle activation: - Supine × 10 reps × (2 sets); - Prone × 10 reps × (2 sets); - On the forearms × 10 reps × (2 sets); - Quadripedal × 10 reps × (2 sets); - On the knees × 10 reps × (2 sets); - On the knee with one knee forward—right and left × 10 reps × (2 sets); - Standing × 10 reps × (2 sets).
Cool-down–stretching exercises.

*Week 2 Exercises.*

Warm-up program
Transition to dynamic phase—while core muscle activation was achieved, the following exercises were applied (Figure A3): - Supine unilateral upper extremity elevation × 10 reps; - Supine bilateral upper extremity elevation × 10 reps; - Supine unilateral hip–knee flexion × 10 reps; - Supine bilateral hip–knee flexion × 10 reps; - Supine contralateral upper lower extremity exercise × 10 reps; - Supine ipsilateral upper lower extremity exercise × 10 reps; - Mini crunches × 10 reps; - Bridge × 10 reps; - Prone scapular adduction × 10 reps; - Prone lie down × 10 reps; - Side-lying hip abduction × 10 reps; - Side-lying hip adduction × 10 reps.
Cool down–stretching exercises.

*Week 3 Exercises*.

Warm-up program
Advanced dynamic phase—while core muscle activation was achieved, the following exercises were applied (Figure A4): - Supine with ball under legs, pushing knees with hands × 10 reps; - Supine with ball under legs, crunches × 10 reps; - Supine with ball under legs, bridge × 10 reps; - Trunk awareness training while lying face down on the ball; - Unilateral upper extremity elevation while lying face down on the ball × 10 reps; - Scapular adduction while lying face down on the ball × 10 reps; - Unilateral hip extension while lying face down on the ball x 10 reps; - Contralateral upper lower extremity exercise while lying face down on the ball × 10 reps; - Prone bridge on hands (plank) × 10 breaths × (5 sets).
Cool-down–stretching exercises.

*Week 4 Exercises*.

Warm-up program
Advanced dynamic phase—while core muscle activation was achieved, the following exercises were applied (Figure A5): - Supine with ball under legs, pushing knees with hands × 10 reps; - Supine with ball under legs, bridge × 10 reps; - Supine with ball under feet, bridge × 10 reps; - Supine on ball, crunches × 10 reps; - Bilateral upper extremity abduction while lying face down on the ball (airplane exercise) × 10 reps; - Bilateral upper extremity flexion while lying face down on the ball (reaching exercise) × 10 reps; - Contralateral upper lower extremity exercise while lying face down on the ball × 10 reps; - Lateral bridge over forearm with knees flexed × 10 breaths × (5 sets).
Cool-down–stretching exercises.

*Week 5 Exercises*.

Warm-up program
While core muscle activation was achieved, the following exercises were applied (dumbbells weighing 1 to 2 kg included) (Figure A6): - Supine with ball under feet, pushing knees with hands × 10 reps; - Supine with ball under legs, crunches with weight in hands × 10 reps; - Supine with ball under legs, bridges with hands extended up × 10 reps; - Sit-ups while lying on the ball × 10 reps; - Bilateral upper extremity abduction with weight in hands while lying face down on the ball (airplane exercise) × 10 reps; - Unilateral upper extremity flexion with weight in hands while lying face down on the ball x10 reps; - Contralateral upper and lower extremity exercises with weight in hands while lying face down on the ball × 10 reps; - Balance and postural awareness training while sitting on the ball; - Unilateral and bilateral upper extremity exercises while sitting on the ball × 10 reps; - Contralateral and ipsilateral upper and lower extremity exercise while sitting on the ball × 10 reps.
Cool-down–stretching exercises.

*Week 6 Exercises*.

Warm-up program
While core muscle activation was achieved, the following exercises were applied (medium-resistance elastic band included) (Figure A7): - Supine with ball under feet, pushing knees with hands × 10 reps; - Supine with ball under legs, pumping hands × 30 breaths; - Supine with ball under legs, straight leg raise in bridge × 10 reps; - Supine bridge with elastic band resistance × 10 reps; - Scapular adduction with weights in hands while lying face down on the ball × 10 reps; - Reaching exercise with weights in hands while lying face down on the ball × 10 reps; - Plank in crawling position without knees touching the ground x 10 breaths × (5 sets); - Bridge on forearms (plank) × 10 breaths × (5 sets); - Open and close the elastic band overhead while standing × 10 reps; - Open and close the band in front of the trunk while standing × 10 reps; - Upper extremity elevation with the band while standing × 10 reps; - Unsupported mini squat × 10 reps; - Unsupported forward lunge × 10 reps.
Cool-down–stretching exercises.

*Week 7 Exercises*.

Warm-up program
While core muscle activation was achieved, the following exercises were applied (advanced phase in postural control, strength, and endurance training) (Figure A8): - Unilateral–bilateral upper extremity flexion with weights in hands while sitting on the ball × 10 reps; - Bilateral upper extremity abduction with weights in hands while sitting on the ball × 10 reps; - Contralateral extremity exercises with weights in hands while sitting on the ball × 10 reps; - Dynamic bridge with arm movements in supine position × 10 reps; - Bridge with weights in hands while supine with hands reaching up to the ceiling × 10 reps; - Lateral bridge (plank) on forearm × 10 breaths × (5 sets); - Scapular adduction with elastic band while standing × 10 reps; - Diagonal upper extremity exercise with elastic band while standing × 10 reps; - Mini squat with weights in hands × 10 reps; - Forward lunge with weights in hands × 10 reps.
Cool-down–stretching exercises.

*Week 8 Exercises*.

Warm-up program
While core muscle activation was achieved, the following exercises were applied (functional training in bipedal stance and final phase with short general repetitions) (Figure A9): - Postural awareness exercise with spinal and pelvic stability while leaning on the exercise ball; - Bilateral upper extremity abduction with weights in hands while leaning back on exercise ball × 10 reps; - Unilateral/bilateral upper extremity flexion with weights in hands while leaning back on exercise ball x 10 reps; - Deep squat with legs abducted while leaning back on exercise ball, with weights in both hands and elastic bands on knees x 10 reps; - Deep squat while lifting exercise ball forward × 10 reps; - Lunge forward while lifting exercise ball forward × 10 reps; - Lift exercise ball up × 10 reps; - Lift exercise ball diagonally and semicircularly × 10 reps; - Side step in deep squat with elastic bands on knees × 10 reps.
Cool-down–stretching exercises.

*Cool-down program:*

Each session ended with a 5 to 10 min cool-down–stretching program (Figure A10):

- Lumbar extensor stretching;
- Iliopsoas stretching;
- Piriformis stretching;
- Hamstring stretching;
- Rectus femoris stretching;
- Hip adductor stretching;
- Upper extremity capsular stretching;
- Pectoral stretching and cervical stretching as needed.
